# Dysregulation of miR-155-5p and miR-200-3p and the Anti-Non-Bilayer Phospholipid Arrangement Antibodies Favor the Development of Lupus in Three Novel Murine Lupus Models

**DOI:** 10.1155/2017/8751642

**Published:** 2017-12-04

**Authors:** Luz Ángela Zárate-Neira, Sandra Sánchez-Barbosa, Abraham Pedroza-Torres, Albany Reséndiz-Mora, Carlos Wong, Isabel Baeza, Carlos Pérez-Plasencia, Carlos Wong-Baeza

**Affiliations:** ^1^Escuela Nacional de Ciencias Biológicas, Instituto Politécnico Nacional, 11340 Ciudad de Mexico, Mexico; ^2^National Institute of Cancer, Tlalpan, 14080 Mexico City, Mexico

## Abstract

Systemic lupus erythematosus (SLE) is characterized by deregulated activation of T and B cells, autoantibody production, and consequent formation of immune complexes. Liposomes with nonbilayer phospholipid arrangements (NPA), induced by chlorpromazine, procainamide, or manganese, provoke a disease resembling human lupus when administered to mice. These mice produce anti-NPA IgM and IgG antibodies and exhibit an increased number of TLR-expressing spleen cells and a modified gene expression associated with *TICAM1*-dependent TLR*-*4 signaling (including *IFNA1* and *IFNA2*) and complement activation. Additionally, they showed a diminished gene expression related to apoptosis and NK cell activation. We hypothesized that such gene expression may be affected by miRNAs and so miRNA expression was studied. Twelve deregulated miRNAs were found. Six of them were common to the three lupus-like models. Their validation by qRT-PCR and TaqMan probes, including miR-342-3p, revealed that miR-155-5p and miR-200a-3p expression was statistically significant. Currently described functions for these miRNAs in autoimmune diseases such as SLE reveal their participation in inflammation, interferon production, germinal center responses, and antibody maturation. Taking into account these findings, we propose miR-155-5p and miR-200a-3p, together with the anti-NPA antibodies, as key players in the murine lupus-like models and possible biomarkers of the human SLE.

## 1. Introduction

Systemic lupus erythematosus (SLE) is a systemic autoimmune disease of unknown etiology characterized by a deregulated activation of T and B cells; polyclonal activation of B cells leads to production of massive quantities of autoreactive antibodies and formation of immune complexes that causes tissue damage. Genetic defects, drug exposure, infectious agents, and environmental factors can also contribute to SLE pathogenesis [1, 2]. Animal models of SLE have been developed and have provided valuable insight into the disease [3]. Our group has described how liposomes with nonbilayer phospholipid arrangements (NPA), induced by either chlorpromazine or procainamide (known for triggering a lupus-like disorder in humans) or by the manganese cation, cause the development of an autoimmune disease resembling human lupus in syngeneic BALB/c and nonsyngeneic NIH mice, along with production of specific anti-NPA IgM and IgG antibodies [4]. A similar disease is produced by directly administering mice with chlorpromazine, procainamide, or manganese (all of which also induce NPA on mice cells) or by injecting the H308 monoclonal antibody, which specifically binds NPA and stabilizes them on mice cells [4, 5]. These findings support the role of NPA for inducing this autoimmune disease in mice, which closely resembles human lupus. NPA induce the production of anti-NPA antibodies, which cause cell damage with the exposure of normally hidden intracellular antigens, such as cardiolipin, histones, nucleus, and DNA with the consequent production of autoantibodies. Interestingly, the anti-NPA antibodies appear 4 weeks before the anti-cardiolipin, anti-histone, anti-nuclear, and lupus anti-coagulant antibodies; it is probable that all these different antibodies contribute towards the establishment of the murine lupus-like and anti-NPA antibodies could have an important role in triggering the production of the other autoantibodies. In fact, we have also demonstrated the presence of anti-NPA antibodies in the sera of patients with SLE or with LES secondary to the antiphospholipid syndrome [4, 6].

Liposomes with NPA also induce TLR-4/MD-2 signaling and, to a lower extent, TLR-2/TLR-6 signaling, both in TLR-expressing HEK cells and bone marrow-derived macrophages (from BALB/c mice) [7]. Mice with lupus-like disease had shown an increase in serum concentrations of proinflammatory cytokines, a higher number of TLR-expressing spleen cells, and an augmented gene expression associated with TICAM1- (TRIF-) dependent TLR-4 signaling (including *IFNA1* and *IFNA2*) and complement activation. In contrast, gene expression associated with apoptosis and NK cell activation was decreased in those mice. This data strongly suggests that TLR-4 and TLR-2/TLR-6 activation by NPA-bearing liposomes triggers an inflammatory response, which in turn leads to production of autoantibodies, as the anti-NPA antibodies, and then, the observed lupus-like disease. Accordingly, we hypothesized that gene expression can be affected by deregulation of some miRNAs, as they have been recently associated with SLE pathogenesis [8, 9]. Micro-RNAs or miRNAs are small noncoding RNAs—22 nucleotides in length approximately—which regulate gene expression at posttranscriptional level. Also, they have been reported as major players in both the physiological control of gene expression and the pathogenesis of malignant, infectious, and autoimmune disorders [10]. Thus, we studied miRNA expression patterns in three versions of our lupus mice model, using liposomes containing NPAs induced by chlorpromazine, promazine, or manganese. The aim of this work was to find key miRNAs associated to lupus-like murine disease and to highlight miRNAs as biomarkers for these murine and human diseases.

## 2. Materials and Methods

### 2.1. Ethics

This study was approved by the Bioethics Committee of our Institution under Protocol ID number CEI-ENCB-025/2014 and complied with the “Guide for the Care and Use of Laboratory Animals” from the US National Institutes of Health [11].

### 2.2. Preparation of Liposomes with and without NPA

L-*α*-Phosphatidylcholine (PC) (Sigma Aldrich, St. Louis, MO, USA) and L-*α*-phosphatidic acid (PA) (Sigma Aldrich) were mixed to form unilamellar liposomes at a 2 : 1 molar ratio. Liposomes were obtained as described previously [4]. Briefly, PC and PA were dissolved in diethylether (JT Baker, Phillipsburg, NJ, USA), TS buffer (Tris-HCl) (Sigma Aldrich) was added, and the mixture was subjected to sonication cycles, diethylether elimination, and 0.45 *μ*m filtration. To induce the formation of NPA in liposomes, liposomes were incubated with 3 mM chlorpromazine (CPZ) (Sigma Aldrich), 8 mM promazine (PZ) (Sigma Aldrich), or 5 mM MnCl_2_ (Mn^2+^) (Sigma Aldrich), respectively, for 30 min at 37°C. The presence of NPA in liposomes was detected by flow cytometry through a previously reported method [4, 5], where liposome complexity is affected by the presence of NPA. FACSCalibur equipment (Becton Dickinson, San Jose, CA, USA) and FlowJo software (FlowJo, LLC, Ashland, Ore, USA) were used for data analysis.

### 2.3. Mouse Model of an Autoimmune Disease Resembling Human Lupus

In previous studies, we described the development of the mouse model of autoimmune disease resembling human lupus [4, 5, 7]. Sixteen female BALB/c mice were obtained from the Laboratory Animal Production and Testing Unit at CINVESTAV-IPN and were housed in the animal facility at our institution. At 2 months of age, mice were divided into groups of four individuals. Three groups were injected with NPA-bearing liposomes induced by chlorpromazine, promazine, or Mn^2+^, respectively, and the other group designated as control was only injected with smooth liposomes without NPA. Groups were injected intrasplenically on days 1 and 15; then, intraperitoneal injections started on day 30 and every week for 1 month more. Blood samples were taken before each intrasplenic injection and every 15 days after intraperitoneal injections started. Serum was separated and then was heated to 56°C for 30 min for complement inactivation. Serum aliquots were frozen at −70°C. All mice were euthanized two months into liposome administration.

Confirmation of disease development was assessed by detecting serum anti-NPA, anti-cardiolipin, anti-histone, and anti-coagulant antibodies, as was previously reported [5]. For anti-NPA antibodies, 96-well flat bottom plates (Costar C, Cambridge, MA, USA) were coated with liposomes with or without NPA (0.1 *μ*mol liposomes in 100 *μ*L TS/well) for 12 h at room temperature. After incubation, plates were blocked 1 h at room temperature with fetal bovine serum (FBS) (200 *μ*L/well of 8% FBS); and afterwards, plates were washed 5 times. Inactivated mice serum was added (100 *μ*L) to each well, and plates were incubated for 1 h at 37°C; then, the plates were washed 5 times. Next, a 1 : 2000 dilution of a goat anti-mouse polyvalent peroxidase-conjugated antibody was used as secondary antibody (100 *μ*L/well, in TS buffer); plates were incubated at 37°C for 1 h and washed 5 times afterwards. Finally, peroxidase substrate 100 *μ*L/well (10 mg *o*-phenylendiamine, Sigma-Aldrich, in 25 mL TS buffer + 20 *μ*L of 30% H_2_O_2_) was added, and after 20 min at 37°C, the reaction was stopped with 2.5 M H_2_SO_4_. Absorbances were read at 492 nm in a Labsystems Multiskan MS (MTX Labsystems, Vienna, VA, USA); each sample was assayed twice.

Anti-cardiolipin, anti-histone, and anti-coagulant antibodies were tested as previously described [4, 12]. ELISA results were reported as arbitrary units for anti-NPA, anti-cardiolipin, and anti-histone antibodies using the ratio (AsP − AsW)/(AsH − AsW), where AsP is the absorbance of sera of mice injected with NPA-bearing liposomes, AsH is the absorbance of serum before liposome injection, and AsW is the absorbance of controls without mice sera [4].

### 2.4. RNA Isolation and miRNA PCR Array Profiling

After 60 days of the first intrasplenical injection of liposomes with or without chlorpromazine-, promazine-, or Mn^2+^-induced NPA, mice were euthanized. Mice spleens were cut into 4 parts, and each was homogenized with 1 mL of TRIzol® reagent (Invitrogen, Carlsbad, CA, USA) plus MagNA Lyser Green Beads (Roche Applied Science, Pleasenton, CA, USA) and was cryopreserved at −70°C until use.

RNA was isolated using the RNeasy mini kit following the manufacturer's protocols (Qiagen, Valencia, CA, USA). Total RNA concentration was determined using a NanoDrop Epoch ND-100 (Thermo Fisher Scientific, Waltham, MA, USA), and RNA samples were quality checked using agarose gel electrophoresis. Sixteen RNA samples (100 ng) were reverse-transcribed with miScript II RT kit (Qiagen), following the manufacturer's instructions.

miRNA arrays were performed by triplicate in 384-well plates from miScript® miRNA PCR array mouse immunopathology kit (Qiagen). This kit was selected for containing primers for 84 well-characterized miRNAs related to immune diseases. Amplification was carried out on a LightCycler 480 (Roche Applied Science), in 40 cycles, as indicated in the manufacturer's protocol. Relative quantification was performed by the ΔΔC_T_ method recommended by the manufacturer. miRNA expression was considered statistically significant if fold change was greater than ± 0.5, as assessed by a two-way analysis of variance (ANOVA; Partek Pro software, Partek Inc., St. Charles, MO, USA) with *p* < 0.05 [13].

We used the miRNA array miScript miRNA PCR array mouse immunopathology kit (Qiagen). This array contains six snoRNA/snRNA (SnoRD61, SnoRD68, SnoRD72, SnoRD95, SnoRD96A, and RNU6-6P) controls for normalization. We choose the RNU6-6P snRNA control for normalization, because its expression was not altered by our experimental conditions.

### 2.5. Array Validation

PCR array results were validated using TaqMan® probes (Applied Biosystems, Thermo Fisher Scientific) for selected miRNAs. Briefly, after miRNA quantification with the StepOne™ system (Applied Biosystems), reverse transcription was performed with 10 ng of total RNA and MultiScribe™ Reverse Transcriptase (RT) (Thermo Fisher Scientific), then inactivated by heating to 85°C for 5 min. The PCR amplification was run as described by the TaqMan Universal PCR Master Mix manual (Applied Biosystems) for the following differentially expressed miRNAs, as determined by PCR array analysis: miR-21a-5p, miR-125a-5p, miR-142-3p/5p, miR-146b/5p, miR-155-5p, and miR-200a-3p. Additionally, miR-342-3p was added because of its importance in human lupus [14, 15], and RNU6 as an endogenous control. All reactions were performed by triplicate in a suitable 48-well plate. Melting curves for specificity were considered, and the relative expression of each miRNA was calculated by the normalized 2^−∆∆CT^ method against RNU6 levels.

Statistical analysis was performed using Prism v6.0 suite (GraphPad software). For group-wise comparisons, each group was normalized against the control mice values and the Holm-Sidak multiple comparison test with *p* < 0.05 was used for determining statistical significance between groups and control, and results were expressed as mean ± SEM.

### 2.6. Target Prediction, Exploratory Analysis, and Signaling Network Development

In order to find the biological significance of the miRNA profiling results, each differentially expressed mice miRNA was researched in the most recent literature. Two online tools for target gene prediction were used: TargetScan v7.0 [16] and miRTarBase v6.0 [17], and those genes predicted by both tools were selected for analysis. The miRTarBase webpage was used to verify correct alignment between selected miRNAs and their corresponding putative target genes.

The signaling network summarizing our current and previous results was developed using Cytoscape v3.4.0 [18] to assess interactions between the mRNA results found in TLR4 signaling pathways [7] and the known targets for miR-155-5p, miR-200a-3p, miR-21a-5p, and miR-146b-5p, trying to find the role of those miRNAs on the development of murine lupus-like disease. These four miRNAs were chosen among the six detected as important in the lupus-like model by miRNA arrays, together with miR-342-3p [14, 15], because they were the only found in the compendium database of experimentally determined miRNA target genes created by the Cancer miRNA Regulatory Network [19]. Using this compendium database, a network was developed to distinguish common targets for these four miRNAs, which were further analyzed with Cytoscape and its associated databases to find relationships among the four miRNAs and their targets, allowing us to highlight only two of the original four miRNAs selected. Afterwards, the final signaling network was specifically compared with TLR4 signaling pathways reported in WikiPathways [20] and recent literature [21–23] to find key players associated with the role of these two miRNAs and our previous data on TLR4 signaling [7].

## 3. Results

### 3.1. Promazine Induces the Formation of NPA in a Similar Way to Chlorpromazine and Mn^2+^

**Figure 1:**
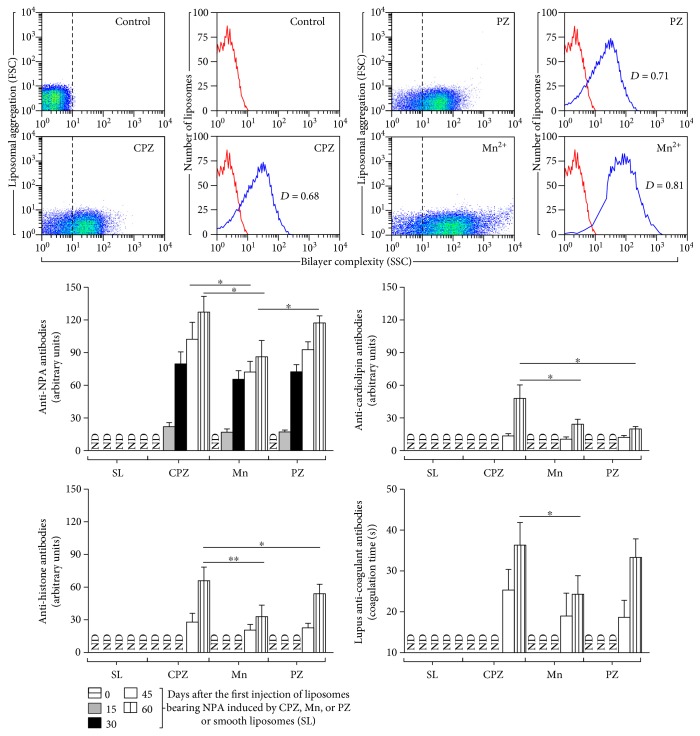
Detection of nonbilayer phospholipid arrangements on liposomes and antibodies on mice sera. Liposomes made of egg-yolk phosphatidylcholine (PC)/egg-yolk phosphatidic acid (PA) (2 : 1 molar ratio) in TS buffer (control) or incubated at 37°C for 30 min with the inductors of nonbilayer phospholipid arrangements: chlorpromazine 3 mM (CPZ), promazine 8 mM (PZ), or manganese 5 mM (Mn^2+^). Changes in bilayer complexity (SSC) are shown as dot plots and histograms: red lines are liposomes alone and blue lines are liposomes incubated with the inductors. Dashed lines indicate reference for changes in bilayer complexity. Kolmogorov-Smirnov test was applied; a statistically significant difference was defined by a *D* value ≥ 0.5. One representative experiment of five is shown. Mice (four per group) were injected with PC/PA liposomes in TS buffer as negative control (SL, smooth liposomes) or liposomes incubated with chlorpromazine, promazine, or Mn^2+^. Anti-non-bilayer phospholipid arrangements, anti-cardiolipin, anti-histone, and lupus anti-coagulant antibodies were measured in mice sera before the injection of liposomes and every 15 days during 60 days after the first injection of liposomes. ND: not detected. Kruskal-Wallis and Dunn tests were applied. ^∗^*p* ≤ 0.05; ^∗∗^*p* ≤ 0.01. Asterisks indicate statistical significance between the antibody titers from the three murine lupus-like models at the indicated time points.

We had previously shown that the presence of NPA can be revealed through flow cytometry by an increased side scatter (SSC) value, in comparison with the SSC value of NPA-free liposomes [4, 5, 24]. Here, we show for the first time promazine as an inductor of NPA in phosphatidylcholine/phosphatidic acid (2 : 1) liposomes (Figure 1 (PZ)), in a similar manner to that previously described for chlorpromazine or Mn^2+^ (Figure 1 (CPZ) and (Mn^2+^)) [5, 7]. As negative controls, liposomes without any inductor were used (Figure 1 (control)).

### 3.2. Promazine-Induced NPAs Produce a Disease Resembling Human Lupus in Mice

Anti-NPA antibodies were detected 15 days after injection of NPA-bearing liposomes induced by promazine. We found titers similar to those from mice injected with chlorpromazine- or Mn^2+^-induced NPAs at the four time points analyzed, except at 60 days when titers of mice injected with chlorpromazine- or promazine-induced NPAs were higher than those of the group injected with Mn^2+^-induced NPA (*p* < 0.05) (Figure 1 (anti-NPA antibodies)). The anti-NPA antibodies, including those produced by promazine-induced NPA, appeared 1 month prior to anti-cardiolipin, anti-histone, and anti-coagulant antibodies (Figure 1 (anti-cardiolipin antibodies), (anti-histone antibodies), and (lupus anti-coagulant antibodies)).

Titers of anti-cardiolipin and anti-histone antibodies produced by promazine-induced NPA were similar to those produced by chlorpromazine- or Mn^2+^-induced NPA. However, at 60 days, the titers of anti-cardiolipin antibodies produced by chlorpromazine-induced NPA had the highest values (*p* < 0.05) (Figure 1 (anti-cardiolipin antibodies)) and the titers of anti-histone antibodies produced by Mn^2+^-induced NPA had the lowest values (Figure 1 (anti-histone antibodies)). Finally, titers of lupus anti-coagulant antibodies produced by promazine-induced NPA were similar to those produced by the other two mice groups, at the two-time points analyzed (Figure 1 (lupus anti-coagulant antibodies)).

Anti-NPA antibodies are strong biomarkers of the murine lupus-like disease resembling human lupus [25], and their presence alongside anti-cardiolipin, anti-histone, and lupus anti-coagulant antibodies confirmed that this autoimmune disease was developed by promazine-induced NPA.

### 3.3. miRNA Profile Highlighted Six miRNAs Differentially Expressed in the Three Lupus-Like Murine Models

**Figure 2:**
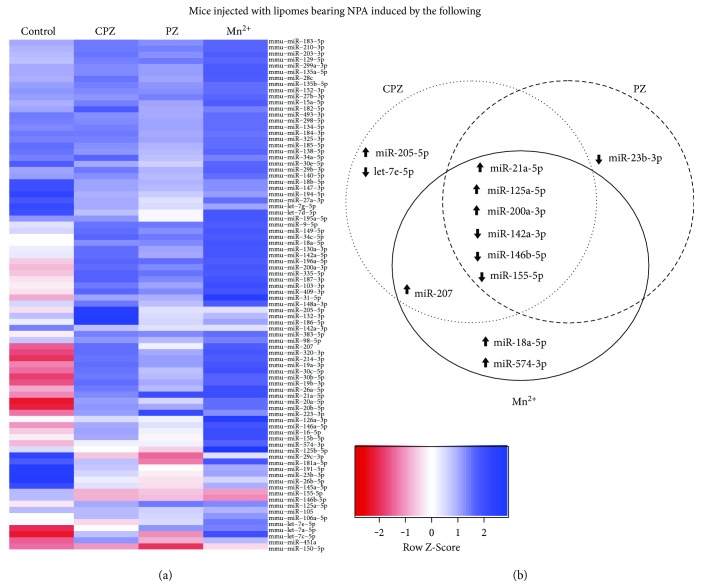
miRNAs obtained by PCR array analysis from the spleens of the three murine lupus-like models. (a) Heat map that indicates down- or upregulation with degrees of red and blue color, respectively. Clustergram at R Studio software was used to compare relative miRNA expression levels in the spleen of mice (four per group) injected with egg-yolk phosphatidylcholine (PC)/egg-yolk phosphatidic acid (PA) (2 : 1 molar ratio) liposomes in TS buffer (control) or liposomes incubated with the inductors of nonbilayer phospholipid arrangements chlorpromazine (CPZ), promazine (PZ), or Mn^2+^. miRNA expression was evaluated with miScript miRNA PCR array mouse immunopathology kit, each row representing an individual miRNA and each column either a murine lupus-like model or control mice. (b) Venn representation of the deregulated miRNAs. The black arrows indicate upregulation or downregulation (inverted arrows) of the corresponding miRNA.

The miRNA profile was obtained through a specialized array that allows the identification of miRNAs, which were previously reported as important in immune diseases, and was able to clearly differentiate the three different mouse lupus models from the control. The differences in expression were easily observed in the generated heat map (representative of three separate experiments) resulting from the 84 miRNAs simultaneously analyzed by this experiment (Figure 2(a)). In total, 12 out of 84 miRNAs showed differential expression in comparison with the control. Six of them were down- (miR-142a-3p, miR-146b-5p, and miR-155-5p) or upregulated (miR-21a-5p, miR-125a-5p, and miR-200a-3p) in the three lupus-like murine models while the other six were affected in two or only one of them (Figure 2(b)). In the lupus-like disease produced by chlorpromazine-induced NPA, let-7e-5p and miR-205-5p were downregulated and upregulated, respectively. In the lupus-like disease produced by Mn^2+^-induced NPA, miR-18a-5p and miR-574-3p exhibited increased expression. Furthermore, both chlorpromazine- and Mn^2+^-derived murine models showed additional upregulated expression of miR-207. Finally, downregulated miR-23b-3p expression was observed in the murine model produced by promazine-induced NPA.

These findings highlight the importance of these miRNAs in the lupus-like murine disease. As expected, the obtained miRNAs have been previously found in human lupus and other autoimmune diseases (Table 1).

### 3.4. miR-155-5p Downregulation and miR-200a-3p Upregulation Seen through miRNA Expression Profiling Were Confirmed by qRT-PCR

**Figure 3:**
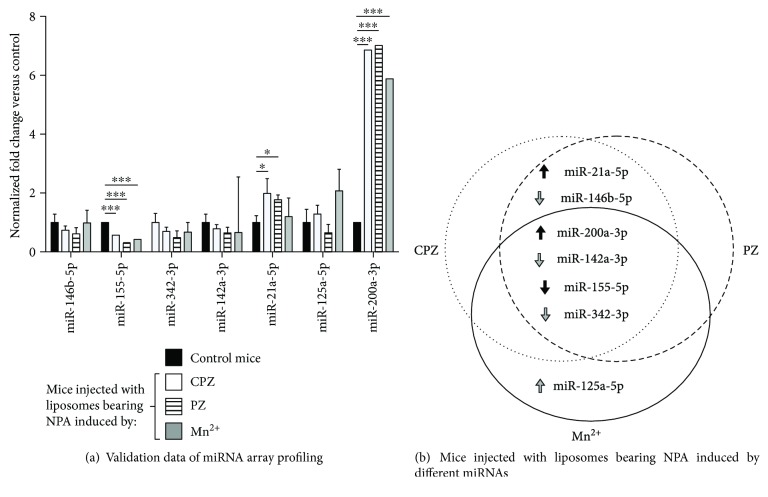
qRT-PCR validation of deregulated miRNAs in the three murine lupus-like models. (a) The validation of the six deregulated miRNAs found by PCR array, together with miR-342-3p because of its importance in human lupus [13, 14], was made using specific TaqMan-directed qRT-PCR. Each miRNA expression level was normalized against endogenous RNU6 by the 2^−∆∆CT^ method and control mice (injected with smooth liposomes). Statistical significance was determined using the Holm-Sidak method. ^∗^*p* ≤ 0.05, ^∗∗∗^*p* ≤ 0.001. Asterisks indicate statistical significance between normalized fold changes from each murine lupus-like model versus the control group. (b) miRNA validation data as a Venn representation. Arrow colors indicate miRNA statistical significance (black for significant and grey for nonsignificant), and their direction shows upregulation or downregulation (inverted).

When deciding to validate only the six miRNAs found to be affected in all lupus-like models tested, we added another miRNA, miR-342-3p, as this miRNA has previously been reported as important in human lupus [14, 15]. A specifically TaqMan-directed qRT-PCR system was selected due to its “gold standard” status for validation of expression data. miR-155-5p showed clearly downregulated expression at the same time as miR-200a-3p showed upregulated expression in all three-murine lupus-like models tested (Figure 3(a)). Also, miR-21a-5p exhibited an upward tendency in expression, but only in the lupus-like disease produced by chlorpromazine- or promazine-induced NPA (Figure 3(a)). Results from the rest of the analyzed miRNAs apparently correspond with the previously observed profiles; however, they were not statistically significant (Figure 3(b) grey arrows).

The downregulation of miR-155-5p and the upregulation of miR-200-3p in the mouse models were detected in the PCR array and with the TaqMan probes, with significant differences compared to the healthy mice. However, no significant differences were found when the expression of these miRNAs was compared between the lupus models. These findings show that there is no significant difference between the miRNA expression profile between the three lupus-like murine models.

**Figure 4:**
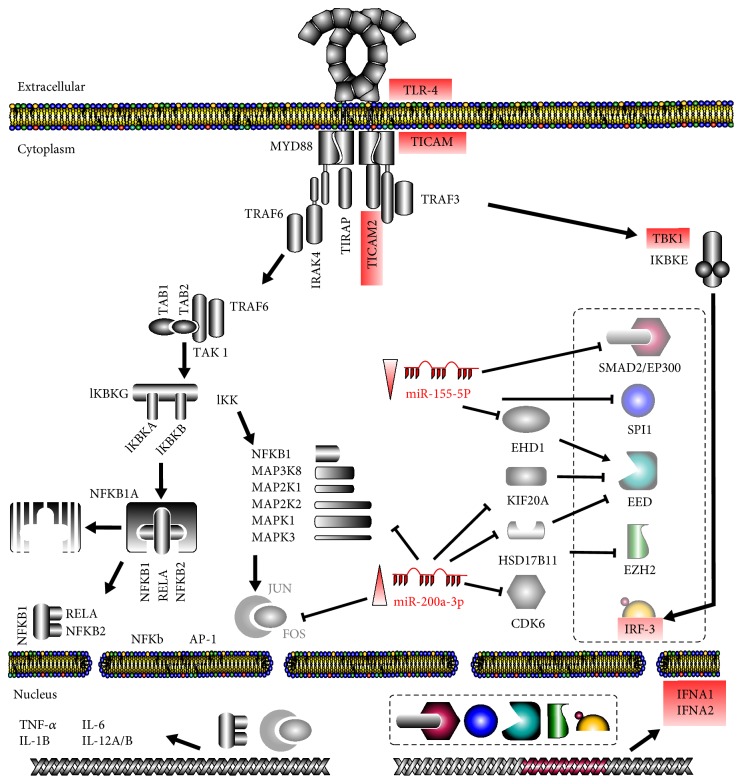
miR-155-5p and miR-200a-3p greatly influence TLR4 signaling pathways. miR-155-5p and miR-200a-3p targets were obtained and analyzed using the database of the Cancer miRNA Regulatory Network [19] along with Cytoscape v3.4.0 [18]. The overall effect of miR-155-5p downregulation and miR-200a-3p upregulation is an increased transcription of *INFA1* and *INFA2*. Here, the two canonical signaling pathways triggered by TLR4 activation are shown, one through *MYD-88* (with the final production of NF*κ*B and AP-1 transcription factors) and the other through *TICAM*s (and production of IRF-3 transcription factor). A close analysis reveals that miR-200a-3p inhibits the production of AP-1 from *MYD*88, downregulating that pathway, while miR-155-5p is positively affecting interferon expression by activating a series of transcription factors. Red boxes indicate our previously described findings [6], and dotted rectangles highlight the transcription factors directly targeted by these miRNA and directly related to interferon gene expression.

Using the database of the Cancer miRNA Regulatory Network [19] and Cytoscape v3.4.0 [18], a signaling network was developed to find relationships among miR-155-5p, miR-200a-3p, miR-21a-5p, and miR-146b-5p and their own targets, which allowed us to highlight only miR-155-5p and miR-200a-3p of the original four miRNAs selected. The signaling network of these miRNAs was specifically compared with the TLR4 signaling pathways previously reported [20]. It was found that miR-155-5p downregulation has a possible positive effect on the production of IFNA1 and IFNA2 because blockage of gene-specific transcription factors *SMAD2*, *EP300*, *SPI1*, and *EED* was removed (Figure 4). In contrast, miR-200a-3p upregulation suppresses gene inhibitors for *EED* and *EZH2* (*KIF20A* and *HSD17B11* for *EED* and *CDK6* for *EZH2*) and allows these two transcription factors to positively affect the transcription of interferons *IFNA1* and *IFNA2*. Interestingly, miR-200a-3p has a direct inhibitory action on *MAPK1* and *JUN*. All these effects of miR-200a-3p on the TLR4 signaling pathway are positive for interferon production, as its upregulation provokes a partial inhibition of the MYD88-dependant signaling branch and diminishes its possible genetic products (e.g., *TNF-α*, *IL-6*, *IL-12A/B*, and *IL-1B*) and helps to activate transcription factors relevant for the interferon-producing TICAM-dependent signaling branch (Figure 4).

## 4. Discussion

SLE is a potentially fatal autoimmune disease characterized by unregulated activation of innate and adaptive immune systems and production of autoantibodies that cause widespread tissue damage [26, 27]. miRNAs, along with epigenetic mechanisms such as CpG-DNA methylation and histone modifications, play a central role in the onset and progression of SLE [28–30].

Because animal models have been used for SLE study, we used the experimental models of lupus developed by procainamide-, chlorpromazine-, or Mn^2+^-induced NPA in order to study the expression profile of miRNAs and its relation with the development of these lupus-like murine diseases, to determine if they could be used as biomarkers for human lupus. In these mice, RNA arrays showed higher expression of genes associated with TICAM1-dependent TLR-4 signaling and the classical complement pathway; mice also showed higher expression of genes related to NOD-2 signaling, antigen presentation and antibody production, and simultaneously, downregulation of genes associated with apoptosis and NK cell activation [7]. We believe that all these results were due to miRNA gene regulation. Here, we evaluated miRNA expression patterns in the lupus-like murine models triggered by promazine-, chlorpromazine-, or Mn^2+^-induced NPA, as possible key players in their pathogenesis.

This work showed, for the first time, promazine as a drug that can elicit a lupus-like disease in mice, as it had also been demonstrated before in humans [31, 32]. Promazine-induced lupus-like disease was similar to that disease already described for chlorpromazine and Mn^2+^, because promazine also induces formation of NPA on liposomes like chlorpromazine or Mn^2+^; in fact, we have demonstrated that these lipid arrangements, NPA, are responsible for inducing the disease [4, 5, 25].

Twelve out of the 84 analyzed miRNAs showed differential expression in the murine lupus-like disease triggered by promazine-, chlorpromazine-, or Mn^2+^-induced NPA. Interestingly, six of these miRNAs showed similar behavior in the three conditions tested, three of them were downregulated (miR-142a-3p, miR-146b-5p, and miR-155-5p) and three were overregulated (miR-21a-5p, miR-125a-5, and miR-200a-3p). The other six miRNAs were affected in two or only one of the three lupus-like models, which showed some differences among these murine models of human lupus. Specifically, two miRNAs (miR-18a-5p and miR-574-3p) were upregulated in the Mn^2+^-induced NPA model, while let-7e-5p was downregulated and miR-205-5p was upregulated in the chlorpromazine-induced NPA model. Furthermore, miR-207 was upregulated in both chlorpromazine- and Mn^2+^-induced NPA models, and miR-23b-3p was only downregulated in the model triggered by promazine-induced NPA.

The six deregulated miRNAs common among the three lupus-like models could be significant contributors to this autoimmune murine disease. They were validated, together with the downregulated miR-342-3p, due to its previously reported importance in LES [14, 15]. Only miR-155-5p and miR-200a-3p were statistically validated (*p* < 0.001) in the three lupus-like models, while miR-21a-5p was validated (*p* < 0.05) only in those triggered by chlorpromazine- or promazine-induced NPA. The analysis of these seven miRNAs and their mRNA targets along with their previously described cell functions in other human and murine diseases leads us to conclude that they participate in inflammatory responses and contribute to triggering lupus-like disease in mice (Table 1).

miR-155-5p is required by B cells for full extrafollicular and germinal center responses. It directly targets *SPI1* and *SHIP-1*, which encode PU.1 and SHIP-1. miR-155-5p underexpression causes an overproduction of PU.1 in B cells, which impairs class-switch DNA recombination and plasma cell differentiation; in consequence, the production of high-affinity IgG antibodies fails. SHIP-1 negatively regulates B cell activation and proliferation [28]. Additionally, miR-155-5p is a negative regulator of *AICDA*, which encodes AID protein, required for promoting somatic hypermutation and the corresponding high-affinity IgG antibody repertoire in antigen-activated B cells [33]. In this way, PU.1 and AID play antagonistic functions: these transcription factors are surely subject to additional regulation that eventually leads to a dominant activity of one of them [34].

It is possible that miR-155-5p underexpression leads to *AICDA* activation in the three murine lupus-like models, mainly due to the known production of specific IgG-class antibodies against NPA in all of these models. Also, it is highly correlated with the higher gene expression related to exogenous antigen presentation and antibody production, as previously found through RNA arrays and confocal microscopy in the murine lupus-like models [7, 25].

miR-200a-3p is overexpressed in SLE [35]. Its overexpression is also related to T helper cell differentiation in multiple sclerosis, possibly by a mechanism involving *SMAD2*, *GATA3*, and *FOXO3* genes, whose participation in Th17 cell differentiation, Treg inhibition, and/or other T helper pathways has been underlined [36]. *SMAD2*, *GATA3*, and *FOXO3* could also be the miR-200a-3p targets in SLE and our lupus-like models. Th17 cell differentiation has been linked to inflammatory processes [37]; in this way, miR-200a-3p overexpression would contribute to the inflammatory response, which leads to the production of IgG antibodies and the observed murine lupus-like disease in our models.

Interestingly, miR-155-5p and miR-200a-3p stand out because of their strong influence on TLR4 signaling, mostly on transcription factors that, in turn, trigger interferon production. Both miR-155-5p and miR-200a-3p cooperate to favor the signaling branch from TLR4 through *TICAM1* and *TICAM2* (*TRAM*) instead of that involving *MYD88*. miR-155-5p underexpression positively affects the production of interferons as it normally inhibits the translation of some transcription factors directly implicated in such production, like *SMAD2*, *EP300*, *SPI1*, and *EED*. Overexpression of miR-200a-3p also contributes positively by inhibiting the TLR4 signaling pathway through MYD88, acting directly on *MAPK1* and *JUN* and interfering with the action of specific inhibitors for transcription factors such as *EED* and *EZH2* involved in the production of interferons IFNA1 and IFNA2. However, further studies on the target genes of miR-155-5 (SMAD2, EP300, and EHD1) and miR-200a-3 (KIF20A, HSD17B11, and CDK6) have to be done.

A closer look into the signaling pathway showed that the overall effect of miR-155-5p downregulation and miR-200a-3p upregulation is an increased transcription of interferons (Figure 4). The importance of interferons was highlighted in our previous results on the TLR4 signaling pathway, where *TLR4*, *TICAM1*, *TICAM2*, *TBK1*, *IRF3*, *IFNA1*, *and IFNA*2 mRNAs showed changes in the murine lupus-like models [7]. Additionally, there are reports of a relationship between interferon production and SLE in humans, where interferon levels are different in different stages of SLE [38, 39]. Single nucleotide polymorphisms possibly affect transcription factor upstream of *IFNA1* [40], and interferon levels are related to autoantibody levels in SLE [41], for example.

In Fas^lpr/lpr^ mice, it was shown that miR155 deficiency decreases the severity of lupus [42], and blocking this miRNA decreases alveolar hemorrhage in mice with Pristane-induced lupus [43]. However, in patients with lupus, miR155 is downregulated [44–46], which correlates with our findings regarding the downregulation of this miRNA in the three mouse models of lupus. Nevertheless, the expression of miR-155-5p and miR-200-3p, together with the anti-NPA antibodies, should be evaluated in other mouse models of lupus and in SLE patients, to validate their possible role as biomarkers of this disease.

The other five miRNAs common on the three models (miR-21a-5p, miR-125a-5p, miR-142a-3p, miR-146b-5p, and miR-342-3p) also have important immunological activities (Table 1) that would together favor antibody production and development of the murine disease.

The remaining six deregulated miRNAs: let-7e-5p, miR-18a-5p, miR-23b-3p, miR-205-5p, miR-207, and miR-574-3p, which are specific to each of our murine lupus-like models, highlight some differences between them, but also show roles on inflammation and immune disease. Among them, let-7e-5p and miR-205-5p, found only in the model produced with chlorpromazine-induced NPA, are known to enhance TLR4 expression (let-7e-5p) [21], provoke *ERBB3* downregulation, and decrease apoptosis (miR-205-5p) [47]. These effects on TLR4 and apoptosis are highly correlated with our previous findings affecting TLR4 signaling and apoptosis in the murine lupus-like model [7].

It can be observed that the twelve deregulated miRNAs, together with miR-342-3p, found in the three-murine lupus-like models, greatly impact the immune system. Interestingly, miR-155-5p and miR-200a-3p, which were statistically validated, play pivotal immune roles and may participate in the development of the lupus-like disease models in BALB/c mice by increasing the production of anti-NPA antibodies, which are responsible for inducing the murine disease. Taking into account these findings and the presence of anti-NPA antibodies both in murine lupus-like and in human patients, we propose that the miRNAs miR-155-5p and miR-200a-3p and the anti-NPA antibodies may play an important role in the development of human LES.

## 5. Conclusions

Two deregulated miRNAs, miR-155-5p and miR-200a-3p, were found to contribute to the development of a murine lupus-like disease triggered by NPA, a recently described molecular association of phospholipids, different from the classical lipid bilayer, and induced by some specific inductors. Our findings support the hypothesis that deregulated miRNAs lead to a proinflammatory environment and, in turn, to an efficient activation of the adaptive immune response with an associated increased IgG antibody production, specifically directed against NPA, and consequently trigger a murine lupus-like disease. Based on this knowledge, we propose miR-155-5p and miR-200a-3p, together with the anti-NPA antibodies, as key players in the murine lupus-like models and possible biomarkers of the human SLE.

## Figures and Tables

**Table 1:** Relevant functions and effects of miRNAs previously described in human lupus and other autoimmune diseases, which were also found deregulated in the three murine lupus-like models.

Data from the three murine lupus-like models analyzed			Data from SLE and other autoimmune diseases		
Deregulated miRNAs	Fold change		Targeted gene	Gene function	Possible effects
	mArray	RTPCR			
▼ miR-155-5p	*0.51*	*0.43*	*SFPI1 (SPI1)*	Impairs class-switch DNA recombination and plasma cell differentiation.	Reduction of B cell activation [28].
			*SHIP-1*	Negative regulator of B cell activation.	Reduction of high-affinity IgG 1 antibodies [33].
			*AICDA*	Higher mutation frequency involved in somatic hypermutation.	B cell activation and production of high-affinity IgG 1 antibodies [34, 48].
			*SMAD2/EP300* ^c^	Transcription factor complex (TFC) triggered by TGF-*β* signaling.	TFC binding to *IFNA1* and *IFNA2* promoter due TGF-*β* activation [48–50].
			*EED* ^c^	Part of a transcription factor complex (TFC).	TFC binding to *IFNA2* promoter [49, 50].

▼ miR-146b-5p	*0.57*	*0.78*	*TRAF6*, *IRAK1*	NF-*κ*B activation.	Increase proinflammatory cytokines (IL-1, IL-6, IL-8, TNF-*α*, and IL-12) [51].
			*STAT1*, *IRF5*	Hyperactivation of type I interferon pathway.	Increase type I IFN (a hallmark of SLE) [52].

▼ miR-142a-3p ▼ miR-142a-5p	*0.51* *0.92*	*0.70* ^a^	*SAP*, *CD84*, & *LL-10*	Increased SAP, CD84, and IL-10 protein levels.	CD4+ T cell overactivation and B cell hyperstimulation [53].

▼ miR-342-3p^b^	*−1.58*	*0.62*	*EP300*, *BMPR2*, & *PDGFRA*	NF-*κ*B activation. Cytokine signaling.	Increase proinflammatory cytokines IL-1, IL-6, IL-8, and TNF-*α* (in type 1 diabetes mellitus) [14, 15]. (miRNA specifically decreased in SLE patients with active nephritis.)

▼ miR-200a-3p	*9.25*	*6.58*	*SMAD2*, *GATA3*, *& FOXO3*	JAK/STAT, TGF-*β*, and mTOR pathways.	Induced differentiation of Th17 cells and inhibited Treg or T helper pathways (in multiple sclerosis) [36].
			*EED & EZH2* ^c^	Part of a transcription factor complex (TFC).	TFC binding to *IFNA2* promoter [49, 50].

▲ miR-21a-5p	*1.16*	*1.65*	*RASGRP1 & DNMT1*	Underexpression of DNA methyltransferase (DNMT1).	Hypomethylation of DNA. A possible key event in the pathogenesis of SLE [54, 55].
▲ miR-125a-5p	*2.90*	*1.34*	*KLF13*	Reduction in the production of *KLF13* and *RANTES.*	Reduction of the inflammatory chemokine RANTES levels [56].

Table 1 summarizes the data previously reported from SLE and multiple sclerosis patients for each miRNA. Direction of triangles indicates upregulation or downregulation (inverted) of miRNAs. ^a^TaqMan probe-primers system used can identify both miRNAs. ^b^miRNA not included in the PCR array, but previously reported as important in lupus [14, 15]. ^c^Data obtained through analysis of miR-155-5p and miR-200a-3p signaling networks by Cytoscape [17] and the experimentally reviewed database from Cancer miRNA Regulating Network [18] (see Figure 4).
